# Tissue Factor Pathway Inhibitor-1 Is a Valuable Marker for the Prediction of Deep Venous Thrombosis and Tumor Metastasis in Patients with Lung Cancer

**DOI:** 10.1155/2017/8983763

**Published:** 2017-01-26

**Authors:** Xianming Fei, Huan Wang, Wufeng Yuan, Mingyi Wo, Lei Jiang

**Affiliations:** Center of Laboratory Medicine, Zhejiang Provincial People's Hospital, Hangzhou 310014, China

## Abstract

Activation of blood coagulation contributes to cancer progression. Tissue factor pathway inhibitor-1 (TFPI-1) is the main inhibitor of extrinsic coagulation pathway. The aim of this study is to assess the predicting significance of TFPI-1 for thrombotic complication and metastasis in lung cancer patients. Total of 188 non-small cell lung cancer (NSCLC) patients were included in this study. Plasma TFPI-1, D-dimer (D-D), antithrombin (AT), Fibrinogen (Fbg), and coagulating factor VIII activity (FVIII:C) were measured. In NSCLC patients, significantly decreased TFPI-1 and AT and increased D-D, Fbg, and FVIII:C levels were observed, and there was a significant correlation between TFPI-1 and other hemostatic parameters (*P* < 0.001, resp.). NSCLC patients with deep venous thrombosis (DVT) or metastasis had significantly lower TFPI-1 levels than those without DVT or metastasis (*P* < 0.01, resp.). Multivariate regression revealed that TFPI-1 acted as a predictor for DVT or tumor metastasis in NSCLC patients [OR: 4.15 or 3.28, *P* < 0.05, resp.]. The area under ROC curve of TFPI-1 was 0.905 (95% CI, 0.842~0.967) or 0.828 (95% CI, 0.742~0.915) for predicting DVT or metastasis (*P* < 0.001, resp.). The optimal point of TFPI-1 was 57.7 or 54.3 ng/mL for predicting DVT or metastasis, respectively. Combination of TFPI-1 and D-D measurements can improve the predicting power for DVT or metastasis in NSCLC patients. Our findings suggested that TFPI-1 was a valuable predictor of DVT and tumor metastasis in NSCLC patients.

## 1. Introduction

Many individuals with cancer are also in a hypercoagulable state [1]. Disorder of blood coagulation and hypercoagulativity may greatly increase the overall risk of thrombosis and even lead to thrombotic events in malignancy patients [2, 3]. Thrombosis is a frequent complication of malignant diseases which is also associated with short survival [4]. Therefore, serious thrombotic complications such as deep venous thrombosis (DVT) are the second leading causes of death in patients with cancer [5]. As we know, lung cancer patients may exhibit more serious disorder of blood coagulation and higher risk of DVT than other malignancy patients. And DVT would more easily result in tumor metastasis and recurrence of lung cancer [6]. Therefore, lung cancer patients have higher incidence of morbidity and mortality [7]. Coagulation inhibitors play important roles in preventing individuals from thrombosis and, at least in part, dysfunctional blood coagulation in cancer patients, which might be the results of inadequate activity of coagulation inhibitors [8].

TFPI, a potent anticoagulant protein, including TFPI-1 and TFPI-2, would inhibit the activity of the TF-factor/VIIa catalytic complex which is involved in activation of IX to IXa and also could directly inhibit FXa which activated blood coagulation. Both endothelial cells and platelets would produce TFPI-1 [9]. Low plasma TFPI-1 levels have been reported in patients with ischemic stroke and thrombotic thrombocytopenic purpura and in women taking combined oral contraceptives [10]. Much studies demonstrated that lower plasma TFPI-1 levels were associated in individuals with venous and arterial thrombotic disease [11]. And tumor cells expressed large quantities of procoagulant molecules in particular tissue factor (TF), which may enhance malignant growth or invasion [12]. But for the inhibitor of TF-factor/VIIa, there were little studies focus on the relationship between TFPI-1 and the progression of lung cancer. Because non-small cell lung cancer (NSCLC) constitutes 85% of lung cancer [13], the aim of this study was to explore the clinical prediction of TFPI-1 for NSCLC patients with DVT and tumor metastasis.

## 2. Materials and Methods

### 2.1. Patients Population

A total of 188 NSCLC patients, including 118 male and 70 female patients aged 41 to 77 years from the Department of Oncology, Zhejiang Provincial People's Hospital, China, between May 2013 and September 2014, were enrolled in our prospective cohort study. All patients were Hans and diagnosed according to the histological diagnosis criteria. The pretreatment evaluation included detailed clinical history and physical examination, the clinical data of patients during hospitalization, and the incidence of DVT formation and tumor metastasis at the time of diagnosis. Patients with DVT or tumor metastasis were screened through either typical or doubtful symptoms before treatment and were confirmed by subsequent compression ultrasonography or histopathology, respectively. The exclusion criteria were primary liver and kidney dysfunctions, after operation, hypertension, cardiovascular and cerebrovascular diseases, complicating other malignancies, inflammation and infections, and other diseases potentially activating blood coagulation system. A disease control group was composed of 112 age and sex-matched patients excluding extra pulmonary diseases especially the diseases mentioned above and only with benign lung diseases including pneumonia, pulmonary tuberculosis, chronic bronchitis, lung sarcoidosis, emphysema, and silicosis. Another 130 healthy individuals (38–80 years) were used as healthy control group. None of the patients and controls were taking any drugs affecting hemostatic and fibrinolytic parameters (e.g., anticoagulating, antiplatelet, and fibrinolytic drugs, and oral contraceptives) in two weeks before samples collecting. Informed consent was obtained from the patients or their relatives, and the study was approved by the institutional review board of Zhejiang Provincial People's Hospital.

### 2.2. Laboratory Analysis

Venous blood of all patients was collected in the morning after an overnight fast to avoid the differences of diurnal variation, especially for hemostatic parameters before treatment, respectively. For coagulation and fibrinolysis, blood sample (9 vol) was collected into vacutainer tubes (Becton Dickinson, Mountain View, CA, USA) containing 0.129 mol/l trisodium citrate (1 vol). Platelet-poor plasma was obtained by centrifugation at 1500*g* at room temperature for 15 minutes. Aliquots of plasma were transferred into plastic tubes without delay and frozen at −80°C until assays for determination of TFPI-1. At the same time, the measurements of plasma D-dimer (D-D), antithrombin (AT), fibrinogen (Fbg), coagulating factor VIII procoagulant activity (FVIII:C), prothrombin time (PT), and activated partial thromboplastin time (aPTT) were performed with a coagulation analyzer (CS-5100, Japan Sysmex) and the commercial reagents (Siemens Healthcare Diagnostics Products GmbH) within 2 hours after samples collection, respectively. The concentration of plasma TFPI-1 was determined using commercially available enzyme-linked immunosorbent assay kit (American Diagnostica). Routine blood and biochemical parameters (including white blood cell and platelet counts, high-sensitive C-reactive protein, and fast serum glucose) were measured. And the biological and clinical data of NSCLC patients (age and sex/Agenda, body mass index, blood pressure, tumor-related, and pathological data) in the duration of hospitalization was collected and reviewed; the incidence of DVT and tumor metastasis at the time of diagnosis was calculated to evaluate the disease conditions of in-hospital NSCLC patients. The fast blood samples of healthy control were collected, and TFPI-1 and other parameters were assayed using the same methods.

### 2.3. Statistical Analysis

Data was analyzed by one way analysis of variance (ANOVA). And statistical analyses were performed by Student's* t*-test for samples of normal distribution data and Mann–Whitney* U*-test for not normal distribution data. And for categorical variables,* Chi-square *test was used. Correlations between the levels of TFPI-1 and other parameters were performed with Pearson correlation analysis. Multivariate Logistic regression analysis was performed to calculate the adjusted odds ratio (OR) and the 95% confidence interval. The receiver operating characteristic (ROC) curve was were constructed, and areas under curves (AUC) were calculated to illustrate the diagnostic power of TFPI-1. *P* value of less than 0.05 was considered statistically significant.

## 3. Results

The clinical characteristics of NSCLC patients were shown in Table 1. Overall, about 54% of the tumors were adenocarcinoma, and 29% were of the type of squamous-cell. At the time of diagnosis, 62.77% of patients had stage IIIb or worse state of NSCLC, and the incidence of DVT and tumor metastasis was 21.80% and 35.11%, respectively. We observed the DVT rate in NSCLC patients with metastasis was significantly higher than those patients without metastasis (*P* < 0.001). The biological and clinical parameters of controls and patients with NSCLC were presented in Table 2. There was no significant difference for levels of the biological parameters in age, gender, smoking status, blood pressure, BMI, Glu, and WBC between the three groups. As expected, CRP level was significantly higher in NSCLC and benign lung diseases groups than that in healthy control group (*P* < 0.05). Compared with the two control groups, Fbg, D-D, and FVIII:C levels were significantly increased in NSCLC patients, whereas AT and TFPI-1 levels significantly decreased (*P* < 0.01). The levels of other parameters including PT, aPTT, WBC, and PLT were not different from the two control groups.

**Figure 1:**
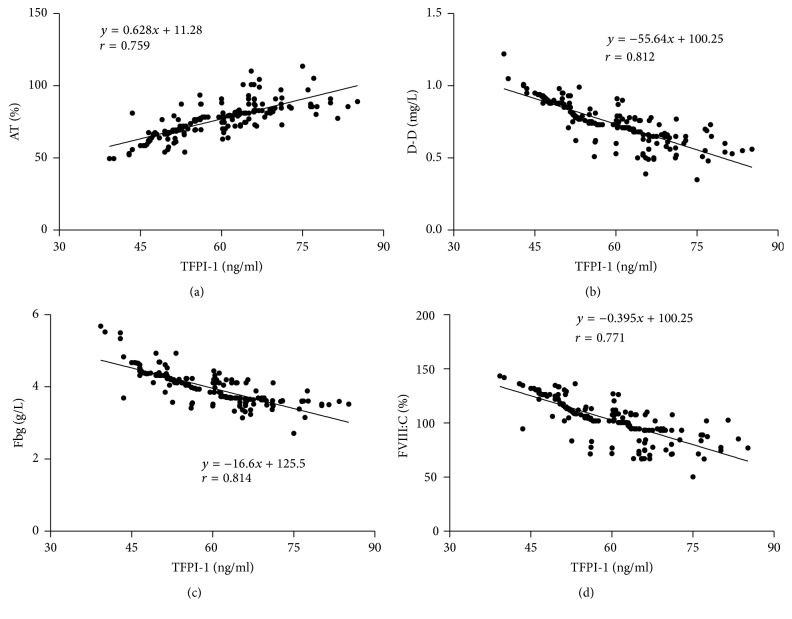
The correlation between plasma TFPI-1 levels and AT, D-D, Fbg, and FVIII:C levels in NSCLC patients. TFPI-1: tissue factor pathway inhibitor-1. AT: antithrombin. D-D: D-dimer. Fbg: fibrinogen. FVIII:C: coagulating factor VIII procoagulant activity.

The correlation between plasma TFPI-1 levels and AT, D-D, Fbg, and FVIII:C levels in NSCLC patients was presented in Figure 1. The plasma TFPI-1 levels were positively correlated with AT levels [*r* = 0.759, *P* < 0.001; Figure 1(a)]. Whereas there was a negative correlation between plasma TFPI-1 levels and D-D, Fbg, and FVIII:C levels [*r*: −0.812, *P* < 0.001;* r*: −0.814, *P* < 0.001;* r*: −0.771, *P* < 0.001, resp.; Figures 1(b), 1(c), and 1(d)].

**Figure 2:**
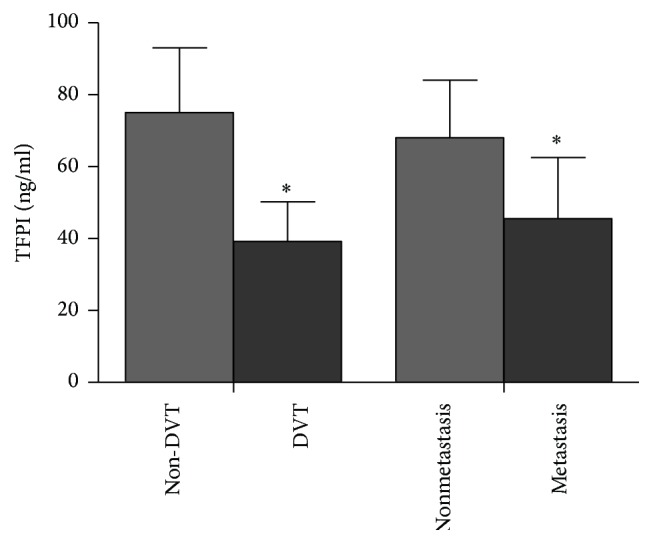
Comparison of TFPI-1 levels in NSCLC patients with different clinical conditions. TFPI-1: tissue factor pathway inhibitor-1. DVT: deep venous thrombosis. ^*∗*^*P* < 0.01: compared with non-DVT and nonmetastasis, respectively.

Comparison of TFPI-1 levels in NSCLC patients with DVT or tumor metastasis with ones without DVT or metastasis was presented in Figure 2. In NSCLC patients with DVT or metastasis, plasma TFPI-1 levels were significantly higher than that in patients without DVT or metastasis (*t*: 24.644, *P* < 0.001;* t*: 20.021, *P* < 0.001, resp.).

Results of multivariate regression analysis of risk factors for complicating DVT or tumor metastasis in NSCLC patients were presented in Tables 3 and 4. Logistic regression analyses showed that TFPI-1 was the independent risk factor related to DVT and tumor metastasis in NSCLC patients (odds ratio, 3.651; 95% confidence interval, 3.011~4.378, *P* = 0.00; odds ratio, 3.122; 95% confidence interval, 2.620~3.781, *P* = 0.00, resp.).

**Figure 3:**
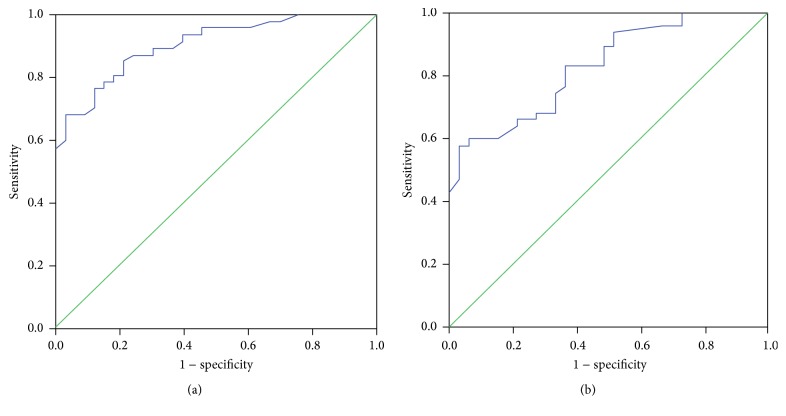
ROC curves of TFPI-1 for predicting DVT and tumor metastasis in NSCLC patients. ROC: receiver operating characteristic. DVT: deep venous thrombosis. (a) ROC curve for DVT. (b) ROC curve for tumor metastasis.

**Figure 4:**
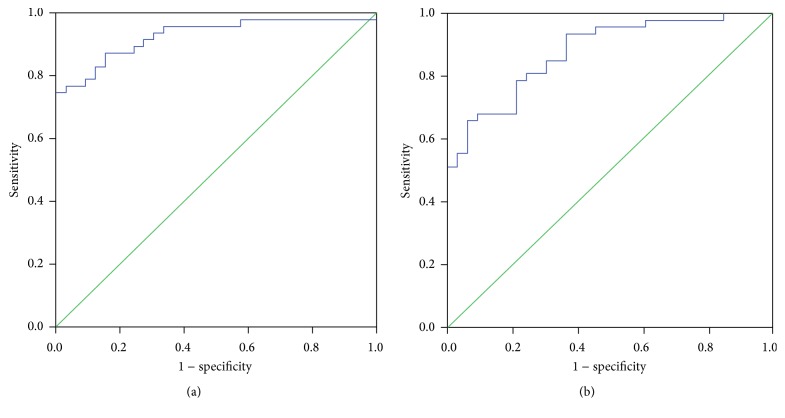
ROC curves of TFPI-1 combining D-dimer for predicting DVT and tumor metastasis in NSCLC patients. ROC: receiver operating characteristic. DVT: deep venous thrombosis. (a) ROC curve for DVT. (b) ROC curve for tumor metastasis.

ROC curves of TFPI-1 for the prediction of DVT or tumor metastasis in NSCLC patients were presented in Figure 3. The ROC curves showed the area under curve of 0.905 (95% confidence interval, 0.842~0.967, *P* < 0.001) for predicting DVT and 0.828 (95% confidence interval, 0.742~0.915, *P* < 0.001) for predicting metastasis. The optimal point (cutoff) of TFPI-1 was 57.7 ng/mL for predicting DVT (sensitivity: 85%, specificity: 68%) and 54.3 ng/mL for predicting metastasis (sensitivity: 80%, specificity: 78%). ROC curves of TFPI-1 combining D-D for the prediction of DVT or tumor metastasis in NSCLC patients were presented in Figure 4. The ROC curves showed the area under curve of 0.928 (95% confidence interval, 0.885~0.985, *P* < 0.001) for predicting DVT and 0.878 (95% confidence interval, 0.805~0.951, *P* < 0.001) for predicting metastasis. The sensitivity was 87% and 85%, and specificity was 79% and 70%, respectively.

## 4. Discussion

Tumor cells may activate coagulation system and lead to thrombosis which is mainly through the production of procoagulant, fibrinolytic, and proaggregating activities and the release of proinflammatory and proangiogenic cytokines [14]. Falanga et al. reported that different components of hemostatic system play an important role in tumor metastasis [15]. Therefore, All of the abovementioned causes may be involved in the occurrence of prothrombotic state even DVT and tumor metastasis in patients with cancer. Some reports have showed that there were higher incidences of DVT, tumor dissemination, and metastasis and higher mortality in patients with lung cancer [14, 16]. In the present study, we found that the incidence of DVT and tumor metastasis before treatment were 21.8% and 35.1% in patients with NSCLC, respectively, which were higher than those 11% or 1~8% reported in other studies [13, 17]. The inconsistent results may be due to difference of population race, the verification of venous thrombosis, and the longer courses of disease before diagnosis of the patients in this study. Another finding was DVT rate of 76% in NSCLC patients with metastasis, and a metastasis rate of about 47% in all patients complicating DVT, indicating that DVT is closely associated with tumor metastasis in NSCLC patients.

Antithrombotic system includes many important components, of which AT is the major anticoagulant in plasma of human body [18]. However, TFPI-1 plays more important roles than AT and other anticoagulants involved in the inhibition of extrinsic pathway of blood coagulation, which is the initiating pathway of blood coagulation [19]. Lower TFPI-1 levels were associated with a lot of thrombotic disease [11]. In the present study, NSCLC patients showed higher D-D and Fbg levels and lower AT levels than that in control individuals. Furthermore, there were lower TFPI-1 levels in NSCLC patients, which indicated that hypercoagulable state existed in NSCLC patients, and decreased TFPI-1 levels were in accordance with abnormal levels of the abovementioned thrombotic and hemostatic parameters. Although the mechanisms by which coagulation is activated in cancer is multifactorial, tissue factor (TF) is traditionally recognized to play an important role in this process [20]. Obviously, as the inhibitor of TF, TFPI-1 is an important inhibitor of coagulation activation. Though we have observed decreased TFPI-1 levels in NSCLC patients, our results seem to disprove the reported results of elevated TFPI in solid tumor diseases [21]. Within our knowledge, the following causes may partly explain the difference. In the present study, only NSCLC were included, and other solid tumors were not. Therefore, different TFPI-1 concentration range might result from different tumor type. TFPI includes both TFPI-1 and TFPI-2 and we did not measure TFPI-2 levels. However, TFPI-2 is a highly effective inhibitor of TF-mediated coagulation and cellular migration [19], which may be an important reason for the difference. We observed the mean of TFPI-1 levels, but it was the median in the report, which may be the third cause of the difference. And our study also demonstrated that TFPI-1 levels were positively and negatively correlated with AT, D-D, Fbg, and FVIII:C levels, respectively, which further indicated that decreased TFPI-1 was closely associated with blood coagulation abnormalities in NSCLC patients, and lower TFPI-1 may have a lower inhibitory power on initial activation of extrinsic coagulation pathway. Therefore, as a result of lower TFPI-1 levels, the risk of thrombotic complications would be increased. Therefore, our study implies that lower TFPI-1 level may lead to the development and progression of the disorder with blood coagulation in NSCLC patients.

Importantly, cancer induced hemostatic activity is a trigger of increased thromboembolic events. On the other hand, DVT has been shown to promote tumor growth and cancer cell dissemination [22]. Some studies have suggested that hemostatic abnormalities observed in cancer patients may lead to the recruitment of inflammatory cells, the generation of tumor stroma, and angiogenesis [23]. For instance, tumors activating coagulation system are supposed to behave more aggressively with higher risk of invasion and metastasis. High levels of circulating biomarkers resembling activated coagulation and fibrinolytic system have been associated with decreased survival for several tumor types [24]. Those studies indicated that progression of disease condition of cancer patients is correlated with abnormal blood coagulation. In the present study, we found that there were significantly lower TFPI-1 levels in NSCLC patients either with DVT or with tumor metastasis than in those patients without DVT or tumor metastasis, respectively. In NSCLC patients with DVT or metastasis, lower TFPI-1 level may be due to consumption by tissue factor from tumor cells or damaged endothelial cells in ongoing coagulation activation. And lower TFPI-1 levels in patients with metastasis may be explained by what TF-VIIa/TFPI-1 interactions which can promote tumor cell adhesion and migration [25]. Through the interactions, TFPI-1 may be partly involved in tumor metastasis and consumed, and lower TFPI-1 was consistent with these processes. Although we could not provide evidences to confirm the direct contribution of lower TFPI-1 level to DVT, the abovementioned significant correlation of decreased TFPI-1 with abnormal hemostatic markers had revealed that lower TFPI-1 level was also closely associated with the occurrence of DVT or tumor metastasis. Moreover, there was a high metastasis rate in patients with DVT and a higher DVT rate in patients with metastasis. Therefore, NSCLC patients with decreased TFPI-1 levels may be in risk for DVT and tumor metastasis, which indicates that TFPI-1 is useful to predict DVT and tumor metastasis at the time of diagnosis of NSCLC.

Various studies have demonstrated that D-D and other markers of coagulation and fibrinolytic activation are strong predictors of poor prognosis for lung cancer [20, 26, 27]. Therefore, the association between TFPI-1 and NSCLC may provide a possible explanation for NSCLC being closely related to DVT, even tumor metastasis. Based on this objective, we evaluated the significance of TFPI-1 for predicting DVT and tumor metastasis at the time of diagnosis of NSCLC. Multivariate regression analysis showed that TFPI-1 had a higher OR value in predicting DVT or metastasis than D-D, Fbg, AT, and FVIII in NSCLC patients, respectively, which indicated that TFPI-1 was a stronger risk factor of DVT and tumor metastasis and also further confirmed that decreased TFPI-1 levels might be correlated with DVT and tumor metastasis in NSCLC patients. Dahm et al. have reported that free TFPI levels below 9 ng/mL were associated with higher risk of DVT in patients with DVT but without known malignancies [28]. Though we only observed total TFPI-1 levels in our study, the findings may indicate the same clinical significance as above mentioned. To further assess the diagnostic power of TFPI-1, ROC curve analysis was used. From the ROC curve analysis, the area under ROC curve (AUC) of 0.884 and 0.856 demonstrated that TFPI-1 is a valuable marker with higher predicting significance for DVT and metastasis at the diagnosis of NSCLC, respectively. And the cutoff values of 57.7 ng/mL and 54.3 ng/mL have a higher sensitivity and specificity in prediction of DVT and tumor metastasis, respectively. High D-D level can predict DVT, and it is also a good prognostic predictor for lung cancer patients [25]. Therefore, we further investigated whether the combination of TFPI-1 and D-D had a higher predictive value for DVT and metastasis in NSCLC patients. As expected, our results demonstrated a higher AUC of 0.928 and 0.878, higher sensitivity of 87% and 85%, and higher specificity of 79% and 70% for predicting DVT and metastasis, respectively, than those of single TFPI-1 measurements, indicating that combining TFPI-1 and D-D measurements may serve as more useful tool in prediction of DVT and metastasis of NSCLC patients.

There are some limitations concerning the present study. Firstly, TFPI-1 detection has no uniform standard and was widely used. We have measured TFPI-1 by ELISA in the study, and the result displayed a valuable clinical significance of TFPI-1 measurements. Furthermore, although we did not measure the direct markers of coagulation activation, we have found that TFPI-1 was significantly correlated to the indirect markers and closely associated with DVT and tumor metastasis, which revealed its clinical predicting values in lung cancer patients. Finally, only symptomatic or doubtful DVT or metastasis patients were included, and we cannot exclude that asymptomatic DVT or metastasis which was present in some patients and potentially decreased the predicting power of TFPI-1.

In conclusion, our study suggests that TFPI-1 is a valuable marker to predict the occurrence of DVT and tumor metastasis with a high sensitivity and specificity at the time of diagnosis of NSCLC patients. Further studies are required to assess whether TFPI-1 could provide aid to the therapeutic decision-making for NSCLC patients.

## Figures and Tables

**Table 1:** The clinical characteristics of patients with non-small cell lung cancer.

	Number (*n*)	Proportion (%)
Number	188	
NSCLC type		
Adenocarcinoma	102	54.25
Squamous cell	55	29.25
Others	31	16.50
Stage of NSCLC		
<IIIb	80	42.55
≥IIIb	108	57.45
Complicating DVT		
Yes	41	21.80
With metastasis	31	75.61
Without metastasis	10	24.39^*∗*^
No	147	78.20
Tumor metastasis		
Yes	66	35.11
With DVT	31	46.97
Without DVT	35	53.03
No	122	64.89

NSCLC: non-small cell lung cancer. DVT: deep venous thrombosis. *P* value: compared with patients with metastasis, respectively. ^*∗*^*P* < 0.001.

**Table 2:** The biological and clinical parameters of controls and patients with NSCLC.

Variables	NSCLC patients	Disease controls	Healthy controls	*P* value
Number of subjects	188	112	130	
Age (years)	45.2 ± 12.3	47.1 ± 11.4	47.9 ± 13.2	>0.05
Sex (M/F)	118/70	61/51	72/58	>0.05
BMI (kg/m^2^)	25.3 ± 2.7	26.1 ± 3.0	25.5 ± 2.7	>0.05
Smoking subjects (M/F)	94/17	45/15	60/17	>0.05
SBP (mmHg)	130 ± 15	132 ± 14	133 ± 12	>0.05
DBP (mmHg)	82 ± 10	80 ± 11	79 ± 12	>0.05
Glu (mmol/L)	5.12 ± 1.55	5.30 ± 1.69	5.25 ± 1.77	>0.05
WBC (10^9^/L)	6.17 ± 2.22	5.74 ± 3.01	5.42 ± 2.15	>0.05
PLT (10^9^/L)	190.1 ± 21.5	199.3 ± 18.0	210.1 ± 20.1	>0.05
CRP (mg/L)	5.5 (0.5–19.5)^△^	4.5 (0.5–14.6)^△^	2.4 (0.5–7.6)	<0.05
PT (seconds)	11.0 ± 3.1	11.6 ± 2.6	11.7 ± 2.2	>0.05
aPTT (seconds)	24.1 ± 5.0	25.8 ± 3.9	26.0 ± 3.8	>0.05
Fbg (g/L)	3.35 ± 1.20^*∗*^	2.68 ± 1.30	2.54 ± 2.01	<0.01
D-D (mg/L)	0.65 ± 0.14^*∗*^	0.42 ± 0.10	0.26 ± 0.04	<0.01
FVIII:C (%)	107.5 ± 12.3^*∗*^	95.1 ± 11.2	91.2 ± 13.0	<0.05
AT (%)	77.0 ± 10.1^*∗*^	92.4 ± 10.8	98.5 ± 13.1	<0.01
TFPI-1 (ng/ml)	51.0 ± 8.6^*∗*^	79.2 ± 13.2	87.1 ± 15.9	<0.01

Data are presented as mean ± SD, and CRP is expressed with median and range. NSCLC: non-small cell lung cancer. BMI: body mass index. SBP: systolic blood pressure. DBP: diastolic blood pressure. GLU: serum glucose. WBC: white blood cell count. PLT: platelet count. CRP: C- reactive protein. PT: prothrombin time. aPTT: activated partial thromboplastin time. Fbg: fibrinogen. D-D: d-dimer. FVIII:C: coagulating factor VIII procoagulant activity. AT: antithrombin. TFPI-1: tissue factor pathway inhibitor-1. *P* value: compared among three groups. ^*∗*^*P* < 0.01: compared with disease controls and healthy controls. *P* < 0.05: compared with healthy controls.

**Table 3:** Multivariate Logistic regression analysis of risk factors for complicating DVT in NSCLC patients.

Variables	Partial regression coefficient	Standard error	Wald *X*^2^	OR (95% CI)	*P*
TFPI-1	1.35	0.55	6.22	4.15 (2.00–8.35)	0.020
AT	1.14	0.38	7.29	3.22 (2.25–5.85)	0.011
Fbg	0.90	0.43	8.01	2.58 (1.15–6.21)	0.004
FVIII:C	0.75	0.50	5.55	1.80 (1.52–6.58)	0.035
D-D	0.56	0.35	10.32	1.35 (1.10–5.22)	<0.001

NSCLC: non-small cell lung cancer. DVT: deep venous thrombosis. TFPI-1: tissue factor pathway inhibitor-1. AT: antithrombin. Fbg: fibrinogen. FVIII:C: coagulating factor VIII procoagulant activity. D-D: D-dimer. OR: odds ratio. CI: confidence interval.

**Table 4:** Multivariate Logistic regression analysis of risk factors for tumor metastasis in NSCLC patients.

Variables	Partial regression coefficient	Standard error	Wald *X*^2^	OR (95% CI)	*P*
TFPI-1	1.72	0.48	9.05	3.28 (2.12–10.98)	0.002
AT	1.40	0.40	7.55	3.00 (2.33–8.65)	0.012
Fbg	1.15	0.60	8.50	2.61 (1.31–8.31)	0.006
FVIII:C	0.90	0.55	6.01	2.02 (1.14–5.11)	0.023
D-D	0.77	0.33	11.39	1.25 (1.07–6.32)	<0.001

NSCLC: non-small cell lung cancer. TFPI-1: tissue factor pathway inhibitor-1. AT: antithrombin. Fbg: fibrinogen. FVIII:C: coagulating factor VIII procoagulant activity. D-D: D-dimer. OR: odds ratio. CI: confidence interval.
